# From Scan to Surgery: Evaluating High-Resolution Computed Tomography (HRCT) and Diffusion-Weighted Magnetic Resonance Imaging (DWI) Accuracy in Detecting Cholesteatoma

**DOI:** 10.7759/cureus.97439

**Published:** 2025-11-21

**Authors:** Aanchal Gupta, G Murugan, A Baskar, Neil A Nunes

**Affiliations:** 1 Radio-Diagnosis, Sree Balaji Medical College and Hospital, Chennai, IND

**Keywords:** cholesteatoma, diagnostic accuracy, diffusion mri, diffusion-weighted imaging, diffusion weighted imaging (dwi), ent infections, high-resolution computed tomography, middle ear, role of mri

## Abstract

Background

Cholesteatoma is a destructive middle ear lesion that frequently leads to bone erosion and complications if not detected early. Accurate imaging is crucial for timely diagnosis and effective surgical planning. High-resolution computed tomography (HRCT) provides excellent anatomical detail, particularly for detecting bony erosions, but it is limited in differentiating cholesteatoma from granulation tissue. Diffusion-weighted magnetic resonance imaging (DWI) has emerged as a non-invasive modality with strong potential for improving diagnostic accuracy. This study aimed to assess the diagnostic performance of HRCT and DWI in identifying cholesteatoma, correlating imaging findings with histopathological examination (HPE) as the gold standard.

Methods

A cross-sectional study was conducted in a tertiary care hospital over 18 months, including 47 patients aged 10-60 years with suspected middle ear lesions. All patients underwent HRCT and DWI, and findings were correlated with HPE results. Clinical and demographic characteristics were analyzed, and diagnostic parameters, including sensitivity, specificity, positive predictive value (PPV), negative predictive value (NPV), and accuracy, were calculated.

Results

Among the 47 patients, 40 (85.1%) were confirmed to have cholesteatoma on HPE. DWI demonstrated perfect diagnostic performance, correctly identifying all 40 cholesteatoma cases and 7 non-cholesteatoma cases, with 100% sensitivity, specificity, PPV, and NPV. HRCT detected bone erosion in 37 patients (78.7%) and demonstrated high sensitivity (91.9%, 34/37) and diagnostic accuracy (80.9%), but specificity was relatively low (40%, 4/10), with moderate NPV (57.1%, 4/7). False positives on HRCT occurred in 6 non-cholesteatoma cases, highlighting its limitations in differentiating soft tissue lesions.

Conclusion

DWI outperformed HRCT in diagnosing cholesteatoma, accurately distinguishing it from postoperative changes and nonspecific inflammatory tissue. While HRCT remains indispensable for assessing bony involvement and surgical mapping, combining HRCT with DWI offers a comprehensive, highly accurate diagnostic approach, improving clinical decision-making, reducing unnecessary second-look surgeries, and optimizing patient outcomes.

## Introduction

The temporal bone is a highly intricate and functionally significant anatomical structure that houses the organs of hearing and balance. It consists of multiple parts-including the squamous, mastoid, tympanic, and petrous portions, that form a protective framework around the delicate auditory and vestibular apparatus. Disorders affecting the temporal bone are common and can arise from congenital malformations, chronic infections, trauma, neoplastic processes, and postoperative changes. These conditions often result in significant morbidity due to their impact on vital sensory functions, making accurate diagnosis and timely management critically important [1].

Over the years, a variety of imaging modalities have been employed to evaluate temporal bone diseases. Conventional options such as plain radiography, air cisternography, and nonionic cisternography were historically used to obtain basic diagnostic information. However, these techniques have important limitations in their ability to visualize the fine structures and subtle pathological changes of the temporal bone. With the advent of advanced cross-sectional imaging, computed tomography (CT) and magnetic resonance imaging (MRI) have emerged as the two most widely used techniques. Together, they have largely replaced earlier methods and are now considered the primary diagnostic tools in clinical practice [2].

High-resolution computed tomography (HRCT) is particularly important in the evaluation of temporal bone disorders. Unlike conventional CT, HRCT uses specialized algorithms that allow for exceptionally fine spatial resolution. This enables detailed visualization of osseous structures within the temporal bone, including the ossicles, semicircular canals, mastoid air cells, and tegmen tympani. The high anatomic accuracy of HRCT facilitates the early detection of pathological changes such as erosions, fractures, and congenital malformations that may not be appreciated on standard imaging. By identifying these abnormalities at an earlier stage, HRCT has significantly improved treatment planning and reduced both morbidity and mortality associated with temporal bone diseases. Additionally, HRCT has allowed clinicians to explore regions that were previously considered “hidden,” thereby enhancing understanding of disease etiology, pathological progression, and surgical anatomy [3].

In contrast, MRI is superior in its ability to visualize soft tissues, vascular structures, and lesions extending beyond bony confines. Because MRI provides excellent contrast resolution, it has broadened the spectrum of temporal bone pathology that can be accurately evaluated [4]. Diffusion-weighted imaging (DWI), an advanced MRI technique, relies on the principle of Brownian motion of water molecules within a voxel. Differences in diffusion characteristics enable the distinction of tissues based on their microstructural properties. This makes DWI especially valuable in clinical settings where otoscopic evaluation is inconclusive or when HRCT results are equivocal. For example, when HRCT is unable to demonstrate subtle middle ear pathology, DWI can assist in confirming the diagnosis of conditions such as cholesteatoma [5].

Cholesteatoma is one of the most significant lesions involving the temporal bone. It is a benign but locally aggressive condition characterized by the abnormal accumulation of keratinizing squamous epithelium and keratin debris within the middle ear cleft and mastoid cavity, often associated with inflammatory responses. Despite its benign histology, cholesteatoma can lead to serious complications, including ossicular destruction, labyrinthine fistula, facial nerve involvement, and intracranial extension, if not diagnosed and treated promptly. HRCT plays a key role in identifying the extent of bony erosions, such as ossicular chain damage, tegmental defects, or lateral semicircular canal fistulae, which are important indicators for surgical intervention [6].

Nevertheless, a key limitation of HRCT is its inability to differentiate cholesteatoma from other nonspecific soft tissue entities, such as granulation tissue, cholesterol granuloma, or postoperative scarring. This diagnostic ambiguity is particularly challenging in previously operated patients, where residual or recurrent cholesteatoma must be distinguished from postoperative inflammatory changes. In such scenarios, DWI has demonstrated a clear advantage. Its ability to detect restricted diffusion makes it highly specific for cholesteatoma, as keratin content exhibits characteristic imaging features that differ from other soft tissue changes [7].

Furthermore, DWI has proven invaluable in postoperative follow-up. In patients who have undergone surgical removal of cholesteatoma, DWI can detect residual or recurrent disease that may not be visible on HRCT. Advances in non-echo planar (non-EPI) DWI techniques have further enhanced this capability by improving lesion detection even when cholesteatomas are small. This technological refinement reduces the need for routine “second-look” surgeries, which were historically performed to confirm or exclude recurrence. By accurately identifying residual disease, DWI minimizes unnecessary invasive interventions and improves overall patient safety and outcomes [8].

Taken together, HRCT and DWI provide complementary information that is critical for the accurate diagnosis and management of temporal bone pathology. HRCT delineates the precise bony anatomy and extent of disease involvement, while DWI distinguishes cholesteatoma from other soft tissue processes and identifies residual or recurrent lesions with high specificity. Their combined use ensures a more comprehensive and reliable evaluation than either modality alone.

Objectives

The objectives of the study included: 1) Evaluating and comparing the diagnostic accuracy of HRCT and diffusion-weighted MRI (DWI) in detecting middle ear cholesteatoma using histopathological findings as the gold standard. 2) Assessing the ability of HRCT to identify bony erosions and ossicular involvement. 3) Determining the role of DWI in differentiating cholesteatoma from postoperative or inflammatory tissue changes. 4) Exploring the combined utility of HRCT and DWI in improving preoperative diagnostic confidence.

## Materials and methods

Study design and setting

This investigation was designed as a cross-sectional study conducted in the Department of Radio-diagnosis and Imaging at Sree Balaji Medical College and Hospital, Chennai. The study was undertaken in a tertiary care hospital setting, where patients are routinely referred for radiological evaluation of suspected middle ear pathologies. Patients were consecutively enrolled during the study period. Those for whom HRCT was initially recommended underwent HRCT first, whereas those for whom MRI was the primary suggested modality underwent MRI initially; thereafter, each patient was evaluated using the alternate imaging modality to allow direct comparison. All imaging studies were independently interpreted by two board-certified MD radiologists, each blinded to the findings and reports of the other. The research period spanned 18 months, from July 2023 to January 2025, allowing adequate time for patient recruitment, imaging evaluation, and correlation with clinical and histopathological outcomes.

Study population and sample size

The study population comprised patients presenting with suspected middle ear lesions or temporal bone pathologies, either newly diagnosed or postoperative cases requiring evaluation for residual or recurrent disease. A total of 47 patients were included in the study, forming a representative cohort for analyzing the complementary diagnostic role of high-resolution computed tomography (HRCT) and diffusion-weighted imaging (DWI).

The sample size was determined using the standard formula for prevalence studies:

$N = \frac{Z_{\alpha/2}^2 \, \sigma^2}{E^2}$

Where Z corresponds to the 95% confidence interval (1.96), P represents the expected proportion in the population (3%) from previous literature [9], α denotes the level of significance, and E is the absolute error (5%). The calculation yielded a required sample size of approximately 46.56, which was rounded up to 47. Thus, 47 patients were enrolled in the study, ensuring adequate statistical power for meaningful analysis.

Inclusion and exclusion criteria

To ensure uniformity, patients were recruited based on well-defined inclusion and exclusion criteria. Individuals between the ages of 10 and 60 years, of either sex, were considered eligible if otoscopic examination revealed a possible middle ear lesion. In addition, patients whose temporal bone HRCT findings were inconclusive, as well as postoperative patients with persistent or recurrent symptoms requiring diagnostic clarification, were included.

Exclusion criteria were applied to ensure patient safety and the reliability of imaging. Patients who declined to provide informed consent were excluded. Pregnant women, cochlear implant recipients, and patients with pacemakers incompatible with MRI were also excluded. Furthermore, patients with any general contraindications to MRI, such as metallic implants or severe claustrophobia, were not considered for recruitment.

Data collection procedure

After receiving approval from the Institutional Ethics Committee, eligible patients were recruited into the study following the provision of written informed consent. Each participant underwent a structured evaluation beginning with a detailed history and semi-structured questionnaire to document demographic details, symptoms, and relevant clinical background (Appendices 1-4).

Following clinical screening, otoscopic examination was carried out by the Department of Otorhinolaryngology to identify possible middle ear lesions. Based on the clinical suspicion, patients were referred for imaging investigations. HRCT of the temporal bones was performed as the initial radiological modality, providing a detailed evaluation of osseous structures and potential erosive changes. Patients were then subjected to an MRI of the temporal bone with DWI sequences, particularly in cases where HRCT findings were inconclusive or when soft tissue characterization was required.

Histopathological findings were retrieved from the Medical Records Department for patients who underwent surgical intervention. These results were used as the reference standard against which radiological findings from HRCT and DWI were correlated.

Imaging techniques and tools

The study utilized modern imaging equipment available at the institution. HRCT of the temporal bone was performed using a SIEMENS SOMATOM go.now 32-slice CT scanner (Siemens Healthineers, Erlangen, Germany). The protocol employed acquisition of images in 5 mm slice thickness, which were subsequently reformatted into 1 mm slices to maximize spatial resolution and allow optimal delineation of fine bony structures such as the ossicles, semicircular canals, mastoid air cells, and tegmen tympani.

MRI examinations were conducted using a GE SIGNA Pioneer 3.0 Tesla scanner (GE Healthcare, USA). The imaging protocol included T1-weighted axial and coronal sequences, T2-weighted axial and coronal sequences, each of 4.0 mm slice thickness, and diffusion-weighted imaging in both axial and coronal planes in 2.0 mm slice thickness. DWI sequences were particularly emphasized for their utility in differentiating cholesteatoma from granulation tissue or postoperative scarring. Lesions demonstrating restricted diffusion appeared hyperintense on DWI and correspondingly hypointense on apparent diffusion coefficient (ADC) maps, consistent with the keratinous nature of cholesteatoma.

Operational definitions

For the purpose of this study, operational definitions of the imaging modalities were established to maintain uniform interpretation. Diffusion-weighted imaging (DWI) was defined as a magnetic resonance sequence that utilizes diffusion-sensitizing gradients to measure the mobility of water molecules within tissues, thereby providing information about cellular density and structural integrity. Cholesteatomas, characterized by restricted diffusion, appeared as hyperintense lesions on DWI images.

High-resolution computed tomography (HRCT) was defined as a CT modality employing thin slices (5.0 mm) and specialized reconstruction algorithms (reformatted to 1.0 mm) to provide high spatial resolution images of the temporal bone. HRCT was considered superior for detecting osseous erosions, ossicular destruction, and structural abnormalities, but was limited in distinguishing between different types of soft tissue lesions.

Statistical analysis

Data collected were systematically entered into Microsoft Excel and subsequently analyzed using Statistical Package for the Social Sciences (SPSS) version 26 (IBM Corporation, Armonk, New York). Descriptive statistics were employed to summarize patient demographics, clinical presentations, and imaging findings. Frequencies and percentages were used for categorical variables, while means and standard deviations were calculated for continuous variables.

The diagnostic accuracy of HRCT and DWI was assessed using sensitivity, specificity, positive predictive value (PPV), negative predictive value (NPV), and overall accuracy, using histopathological examination as the gold standard. Comparative analysis of HRCT and DWI findings was performed to assess their complementary diagnostic roles, particularly in differentiating cholesteatoma from other nonspecific tissue changes.

## Results

Among the 47 patients included in the study, the majority were aged above 40 years (n=16, 34%), followed by those aged 10 years (n=12, 25.5%). Smaller proportions were observed in the 11-20 years (n=7, 14.9%) and 31-40 years (n=7, 14.9%) categories, while the least representation was in the 21-30 years group (n=5, 10.6%). Males constituted a higher proportion of the study sample (n=30, 63.8%) compared to females (n=17, 36.2%). With respect to surgical history, 12 patients (25.5%) had undergone previous surgery, whereas 35 patients (74.5%) presented without any surgical history (Table 1).

**Table 1:** Demographic and Clinical Characteristics of the Study Population (n=47)

Characteristics	Frequency (n)	Percentage (%)
Age (years)		
10	12	25.5
11–20	7	14.9
21–30	5	10.6
31–40	7	14.9
>40	16	34.0
Gender		
Male	30	63.8
Female	17	36.2
History of Surgery		
Yes	12	25.5
No	35	74.5

On high-resolution computed tomography (HRCT), bone erosion was identified in 37 out of 47 patients (78.7%), whereas 10 patients (21.3%) showed no evidence of erosion. Diffusion-weighted imaging (DWI) revealed restricted diffusion in 40 patients (85.1%), while only 7 patients (14.9%) demonstrated no restriction. These findings indicate that both HRCT and DWI provided significant diagnostic yield in the evaluation of suspected middle ear lesions (Table 2).

**Table 2:** Distribution of Patients Based on HRCT Bone Erosion and DWI Findings (n=47) HRCT - High-resolution computed tomography, DWI - Diffusion-weighted imaging

Imaging Findings	Frequency (n)	Percentage (%)
Bone Erosion on HRCT		
Yes	37	78.7
No	10	21.3
DWI Findings		
Restriction	40	85.1
No Restriction	7	14.9

In the present study, Table 3 shows that most lesions demonstrated T1 hypointensity in 31 patients (66.0%), while 4 patients (8.5%) showed T1 hyperintensity and 2 patients (4.3%) were isointense. On T2-weighted imaging, all lesions (47/47, 100%) appeared hyperintense, highlighting the uniformity of T2 signal characteristics in the study population. These findings emphasize that while T1 signal characteristics varied, T2 hyperintensity was a consistent feature across all cases.

**Table 3:** Distribution of T1 and T2 MRI Signal Characteristics among Study Population (n=47) T1WI - T1 weighted image, T2WI - T2 weighted image

MRI Findings	Frequency (n)	Percentage (%)
T1WI Characteristics		
Hyperintense	4	8.5
Hypointense	31	66.0
Isointense	2	4.3
T2WI Characteristics		
Hyperintense	47	100

Table 4 shows the diagnostic accuracy findings among the tests. When comparing imaging findings with histopathological examination (HPE), diffusion-weighted MRI (DWI) demonstrated 100% sensitivity, specificity, and positive predictive value (95% CI: Sensitivity 91.24-100, Specificity 64.57-100), indicating its robust diagnostic accuracy for detecting cholesteatoma. In contrast, high-resolution computed tomography (HRCT) showed a sensitivity of 91.89% (95% CI: 78.7-97.2) but a relatively low specificity of 40% (95% CI: 16.82-68.73). The positive predictive value of HRCT was 85%, while the negative predictive value was only 57.14%. The overall diagnostic accuracy of HRCT was 80.85% (95% CI: 67.46-89.58). These findings highlight that although HRCT is highly sensitive for detecting bony erosions and lesions, DWI provides superior diagnostic accuracy in differentiating cholesteatoma from other postoperative or inflammatory changes.

**Table 4:** Diagnostic Accuracy of DWI and HRCT Compared to Histopathological Examination (HPE) among Study Population (n=47) DWI - Diffusion-weighted imaging, HRCT - High-resolution computed tomography

Parameter	DWI (%) (95% CI)	HRCT (%) (95% CI)
Sensitivity	100 (91.24 – 100)	91.89 (78.7 – 97.2)
Specificity	100 (64.57 – 100)	40.00 (16.82 – 68.73)
Positive Predictive Value	100 (91.24 – 100)	85.00 (70.93 – 92.94)
Negative Predictive Value	100 (64.57 – 100)	57.14 (25.05 – 84.18)
Diagnostic Accuracy	100 (92.44 – 100)	80.85 (67.46 – 89.58)

## Discussion

The present study explored the demographic, imaging, and histopathological characteristics of patients with suspected cholesteatoma, focusing on the diagnostic performance of HRCT and DWI-MRI. The age distribution revealed a bimodal pattern, with peaks in early childhood (0-10 years, 25.5%) and later adulthood (>40 years, 34%). This finding suggests that cholesteatoma can affect both pediatric and adult populations, potentially reflecting congenital as well as acquired etiologies. The mean age was 30.3 years (SD 18.5), with a range from 10 to 60 years, highlighting the broad age group affected. Previous studies have also shown a similar distribution, with Janarthanan et al. reporting substantial cases in the 11-20 years and 31-40 years groups, supporting the observation that cholesteatoma occurs across age ranges with no single predominant demographic [10].

Gender analysis showed a male predominance, with 63.8% of cases being male compared to 36.2% female. This finding may point toward a true male predisposition to the disease or could be attributed to sociocultural and healthcare-seeking factors influencing hospital attendance. Janarthanan et al. also reported a similar pattern, with a higher number of males diagnosed with cholesteatoma [10]. Such consistency across studies strengthens the evidence of gender disparity, though biological, environmental, and behavioral factors may all contribute.

In terms of prior surgical history, 25.5% of patients had undergone surgery, while the majority (74.5%) had not. This suggests that while recurrence or complications following surgery remain an important issue, most cases in this cohort were primary presentations. Including both primary and recurrent cases provided a comprehensive overview of disease manifestation.

HRCT findings revealed bone erosion in 78.7% of patients, a strong indicator of the aggressive and destructive potential of cholesteatoma. Bone erosion is often a hallmark of advanced disease and significantly impacts surgical planning. In this study, the presence of bone erosion showed a high sensitivity of 91.89% for cholesteatoma, but specificity was only 40%, reflecting that HRCT can sometimes give false positives due to other causes of erosion, such as granulation tissue or chronic infection. The diagnostic accuracy of HRCT was 80.85%, showing it to be a useful but imperfect diagnostic tool. This aligns with previous reports by Janarthanan et al., who documented HRCT sensitivity of 81.25% and specificity of 83.3% [10]. Similarly, Xun et al. reported HRCT sensitivity and specificity values of 0.68 and 0.78, respectively, confirming that while HRCT provides an excellent anatomical roadmap, it cannot alone confirm the diagnosis of cholesteatoma [11]. Ayyaril et al. further emphasized HRCT’s role in localizing the lesion with 96.6% accuracy, but suggested that combining HRCT with DWI could yield superior diagnostic outcomes [12]. Naveen et al. demonstrated that HRCT had a sensitivity and specificity of 83.3% and 58.3%, respectively, supporting the current findings that HRCT can be sensitive but lacks specificity [13]. Shreelatha et al. also highlighted that HRCT performed better in primary cases (87.5%) but poorly in recurrent/residual disease (25.0%), suggesting limitations in certain clinical contexts [14].

DWI findings showed that 85.1% of patients demonstrated restricted diffusion, and this correlated perfectly with histopathology. All 40 patients with restriction had cholesteatoma confirmed by HPE, and all 7 without restriction did not have cholesteatoma, resulting in 100% sensitivity, specificity, PPV, and NPV. This demonstrates the remarkable diagnostic accuracy of DWI, highlighting its role as a non-invasive and highly reliable tool. These findings mirror those of Janarthanan et al., who reported identical diagnostic performance with 100% sensitivity, specificity, PPV, and NPV [10]. Xun et al. reported slightly lower values, with a sensitivity of 0.88 and specificity of 0.93, but still emphasized DWI’s superior performance compared to HRCT [11]. Aikele et al. also found high diagnostic accuracy, with a sensitivity of 77% and a specificity of 100%, reinforcing that while occasional false negatives can occur, DWI is generally highly reliable [15]. Ayyaril et al. reported sensitivity of 100% and specificity of 80%, again supporting the high value of DWI in clinical practice [12]. Naveen et al. reported sensitivity and specificity of 91.6% each, while Shreelatha et al. demonstrated 94.23% sensitivity and 100% specificity, both consistent with the current results [13,14].

The study also assessed MRI signal characteristics, showing that 66% of lesions were hypointense on T1, 8.5% hyperintense, and 4.3% isointense. These findings are in line with the established profile of cholesteatoma, where hypointensity on T1 is common due to keratin debris. On T2 imaging, 100% of lesions were hyperintense, reflecting the fluid or keratin-rich content. Although these features are not highly specific, they add to the diagnostic profile when combined with DWI. The mean ADC value was 1.5 (SD 0.5), which supports restricted diffusion in cholesteatoma. Ayyaril et al. had reported ADC values below 1.226 × 10⁻³ mm²/s as being predictive of cholesteatoma, highlighting the utility of quantitative assessment in challenging cases [12].

Histopathology confirmed cholesteatoma in 85.1% of patients, with granulation tissue in 10.6% and inflammation in 4.3%. This emphasizes that while cholesteatoma is the dominant pathology in such cases, mimicking conditions are also common. Stefanescu et al. reported that pars flaccida cholesteatoma accounted for 35% and pars tensa for 19% of cases, while scutum and incus erosion were seen in 70% and 67% of patients, respectively, underscoring the destructive potential of the disease [16].

When correlating HRCT and HPE findings, 85% of patients with cholesteatoma had bone erosion, whereas erosion was also seen in 3 out of 7 non-cholesteatoma patients. This highlights HRCT’s limitation, as it may overestimate disease by showing erosion due to other causes. In contrast, DWI showed a perfect correlation with HPE, with no false positives or negatives. These findings confirm that while HRCT is invaluable for surgical planning and assessing the extent of disease, DWI is superior for diagnostic confirmation. Multiple studies, including those by Schwartz et al., Constable et al., and Kimitsuki et al., support the conclusion that a combination of HRCT and DWI provides the most comprehensive preoperative evaluation, balancing anatomical detail with diagnostic accuracy [17-19].

The findings of this study must be considered in light of certain limitations. First, the relatively small sample size (n=47) limits the generalizability of the results, particularly for subgroup analyses such as recurrent versus primary disease or pediatric versus adult populations. Second, the study was conducted in a single center, which may introduce referral and selection biases. Third, although DWI showed perfect diagnostic accuracy in this cohort, confidence intervals for specificity and NPV were relatively wide due to the smaller number of negative cases. Finally, the cost and availability of MRI may limit the widespread applicability of DWI in resource-constrained settings.

Despite these limitations, the study carries important implications. The results strongly emphasize the pivotal role of DWI-MRI as a highly reliable and non-invasive imaging technique for diagnosing cholesteatoma, demonstrating near-perfect sensitivity and specificity. HRCT remains indispensable for surgical planning due to its superior depiction of bone erosion and anatomical detail, though it should not be used alone for diagnostic purposes. In combination, HRCT and DWI offer a comprehensive evaluation framework that enhances both diagnosis and management. Future large-scale, multicentric studies are warranted to validate these findings, assess cost-effectiveness in routine practice, and evaluate their influence on surgical outcomes. Integrating DWI into standard diagnostic workflows could help reduce unnecessary surgeries, enable timely intervention, and ultimately improve patient outcomes.

## Conclusions

In conclusion, this study demonstrates that diffusion-weighted MRI (DWI) provides excellent diagnostic accuracy for cholesteatoma, showing 100% sensitivity, specificity, and predictive values in this cohort when compared with histopathology. These findings reaffirm DWI as a highly reliable, non-invasive imaging technique for the evaluation of suspected cholesteatoma. However, the near-perfect performance observed should be interpreted with caution, as spectrum and verification biases, single-center design, and limited sample size may influence the results. Nonetheless, very small residual or recurrent lesions, postoperative artifacts, and limited MRI accessibility in resource-constrained settings remain practical challenges that could affect real-world applicability.

While HRCT continues to be indispensable for assessing bony erosion and anatomic detail, it should ideally be complemented by DWI to achieve a comprehensive preoperative evaluation. Future multicentric studies with larger, more diverse cohorts, standardized imaging protocols, and longitudinal follow-up are warranted to validate these findings, assess cost-effectiveness, and refine diagnostic algorithms. Integrating optimized DWI protocols into standard clinical workflows holds promise for improving diagnostic precision, minimizing unnecessary surgical exploration, and enhancing patient outcomes.

## Figures and Tables

**Figure 1:**
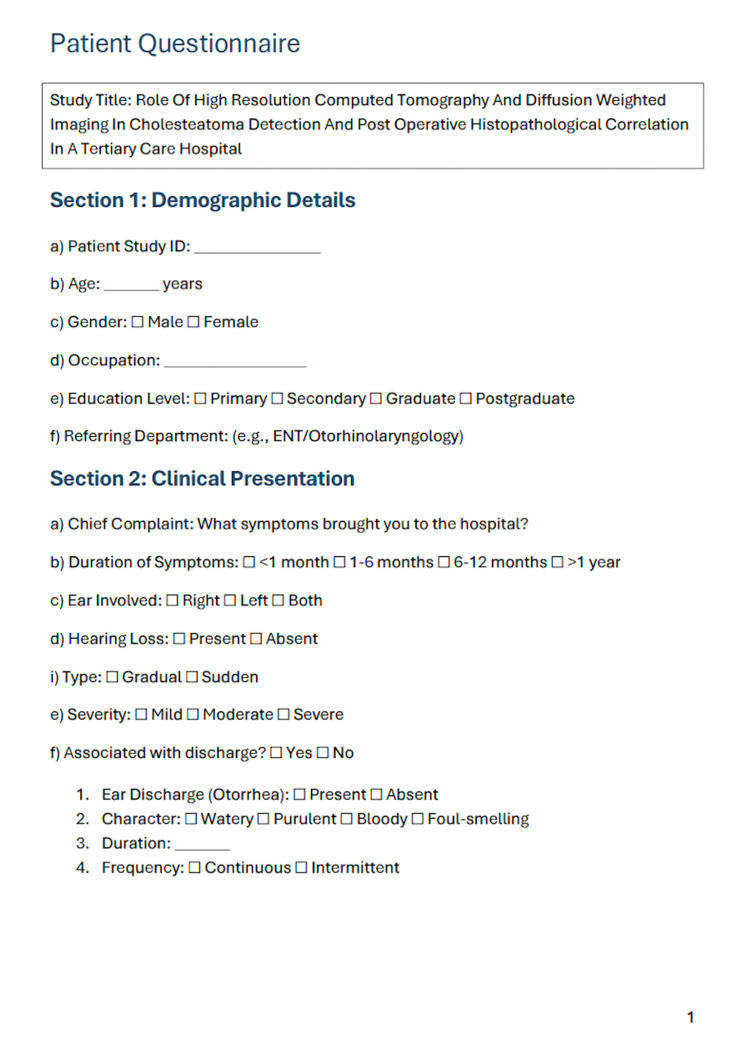
Patient Questionnaire - Part 1

**Figure 2:**
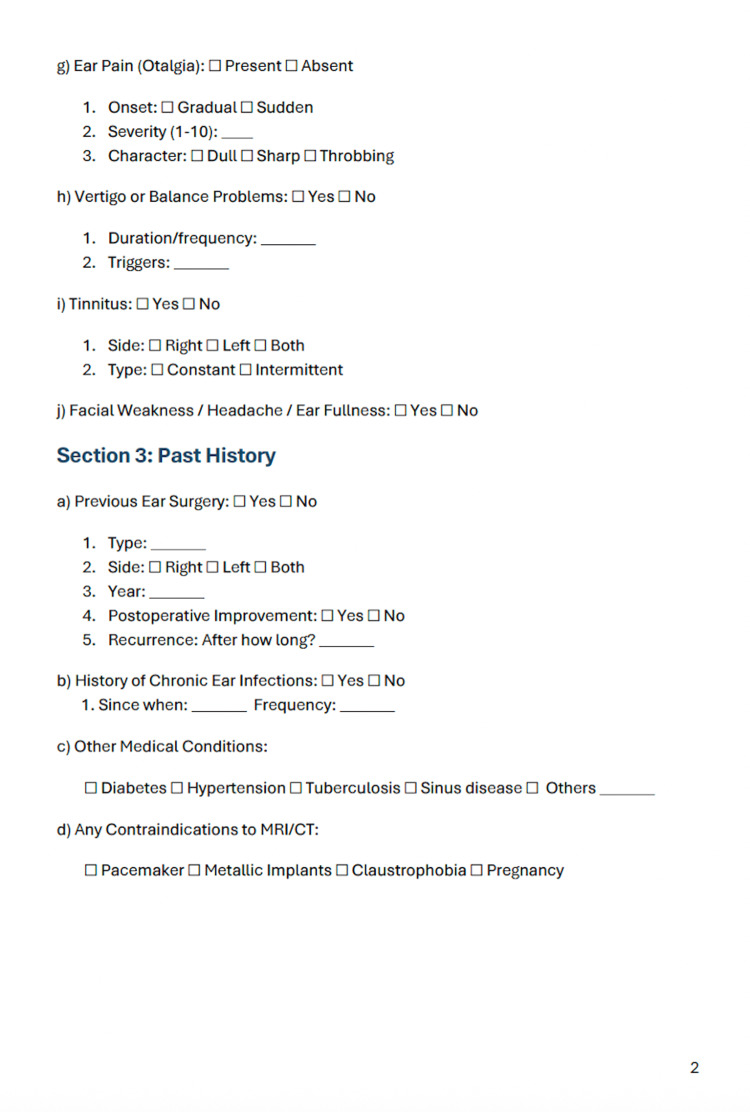
Patient Questionnaire - Part 2

**Figure 3:**
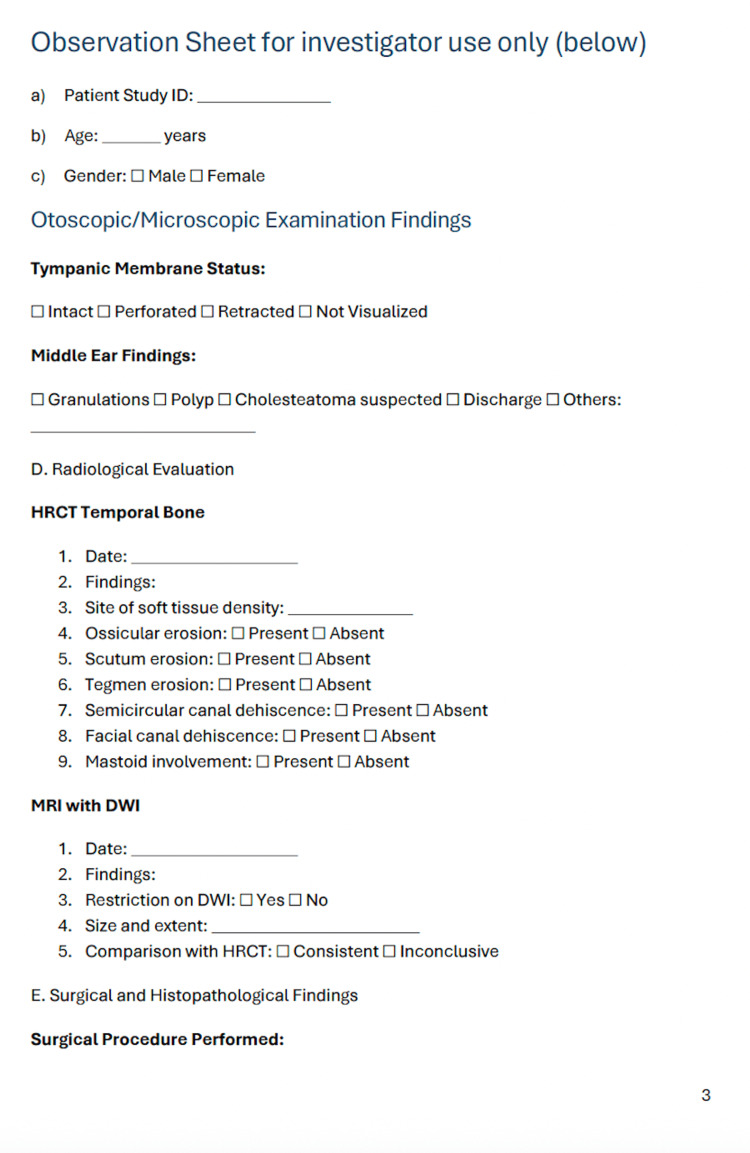
Patient Questionnaire - Part 3

**Figure 4:**
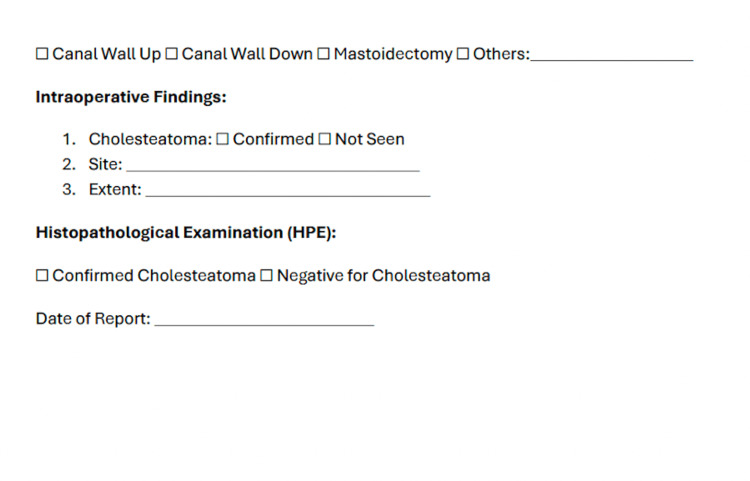
Patient Questionnaire - Part 4

## Appendices

Appendix 1 

Appendix 2 

Appendix 3 

Appendix 4 
